# The timing of energy allocation to reproduction in an important group of marine consumers

**DOI:** 10.1371/journal.pone.0199043

**Published:** 2018-06-27

**Authors:** Blaine D. Griffen

**Affiliations:** Department of Biology, Brigham Young University, Provo, Utah, United States of America; Department of Agriculture and Water Resources, AUSTRALIA

## Abstract

Organisms may energetically finance reproductive effort using energy stored prior to the reproductive period (termed capital breeders) or using energy acquired during the reproductive period (termed income breeders). The specific strategy used has implications for population dynamics as well as for the response to environmental variation. Crabs and other crustaceans have generally been assumed to be capital breeders. Here I demonstrate an experimental procedure used to determine whether crabs are capable of using an income breeding strategy. I then examine data from several published studies from a range of crab species across a broad phylogenetic spectrum that conducted similar experiments to look for evidence of income breeding strategy. I show that income breeding does occur in crabs, but that it appears to be taxon-specific. In particular, I show that income breeding occurs in two species from the family Portunidae, but fail to find evidence for income breeding in other taxa examined. This finding has a range of implications for this ecologically and economically important group of consumers, including implications for their response to human-induced environmental change, their response to fishing pressure, and best practices for aquaculture. The implications of breeding strategy in crabs likely depends on phylogeny (morphology) and ecology, both of which influence the space available for energy storage inside the carapace.

## Introduction

The energetics of individual organisms play an important role in ecological processes, determining, for instance, where organisms can survive [1], the allocation of time to different activities [2], the rate at which organisms grow [3], the timing of important life-history processes [4], and the timing and level of reproductive performance [5]. The timing of energy and nutrient allocation towards reproductive investment varies across organisms. Some store energy used in reproduction prior to the start of the reproductive process, termed capital breeders, while others finance reproduction using energy gained throughout the reproductive process, termed income breeders [6,7]. While these two possibilities were originally viewed as alternative strategies [8,9], they are now more appropriately viewed as endpoints of a continuum, with many organisms showing mixed capital-income strategies [10], and organisms falling at different places along this continuum.

A species’ location along the capital-income continuum has important implications for population growth and dynamics. For instance, species that rely more on energy capital built up over long periods are expected to display more pronounced time lags than income breeders in population response to environmental variation [11]. Empirically, the timing of energy storage for reproduction has been linked to population cycling and explosive population growth [12] and to the occurrence of carry-over effects as individuals move from one location (feeding area) to another (breeding area) [13].

Where animals fall along the capital-income continuum also has important expected implications for their ability to withstand many of the environmental changes being imposed by human impacts today [11]. Common human-induced environmental changes such as climate change, habitat destruction, or pollution can lead to reduced habitat quality. And reduced habitat quality can in turn lead to reduced body condition [14,15], which may subsequently hamper reproductive performance [16,17]. Additionally, energy storage has an ecological cost in terms of increased metabolism [18], and the metabolic costs of energy storage involved in capital breeding should exceed those for income breeders [19]. And while the energetic cost of capital breeding was previously assumed to be minimal for small bodied ectotherms [20], recent work demonstrates that differences in energy storage in these animals may account for more than a quarter of the variation in metabolic costs [21]. The impacts of these increased metabolic costs may be especially pronounced when environments deteriorate and the opportunities for energy/nutrient acquisition are compromised. Thus, determining where animals fall along the capital-income continuum is an important step in understanding and predicting the responses of different groups of organisms to the rapid environmental changes now taking place.

A group of organisms that are experiencing a shift in their ecological role as a result of human-induced environmental change are the brachyuran crabs. Brachyuran crabs, along with other crustaceans, play a central ecological role in marine benthic habitats [22] where they may drive trophic cascades [23–25] through both their consumptive and nonconsumptive interactions [26]. Further, their role as top consumers in near-shore food webs is becoming increasingly pronounced with fishery-induced removal of large predatory fish [27,28]. In addition, they are important economically, supporting active fisheries and aquaculture enterprises around the world, a role which is similarly becoming increasingly important with the depletion of wild fish stocks [29,30].

In addition, crabs themselves are often the agent of environmental change via their role as highly successful invaders. Crabs are extremely resilient [31] and are thus commonly able to survive transport (in ballast, in seafood packaging, etc.) and to successfully colonize new environments [32]. As a result, at least 73 crab species have been introduced to regions around the world outside their native range, and 48 of these (65.8%) have become successfully established [33]. Further, many of these species have now successfully invaded multiple times on different continents [34,35]. In addition to their role as prominent invaders, crabs can also affect environmental change via range expansion. For many of the same reasons regarding crab resiliency, systems examined thus far suggest that crabs are capable of responding to climate change with rapid range expansions, often into novel environments where they alter community dynamics [36–38].

As has been previously discussed [11], there is no single time period for measuring or distinguishing capital and income breeding that is appropriate for all species and life history strategies. Consequently, it is necessary for each individual study to define the period of interest, justifying the periods chosen based on the biology of the species examined [11]. Here I delineate capital and income periods of breeding by the point at which energy begins to be allocated specifically for reproduction. Crabs energetically invest heavily in reproduction during the process of oocyte production and vitellogenesis (reviewed in [39]). The duration of vitellogenesis can vary substantially between species, but commonly can be separated into a slow phase of gradual buildup (Vitellogenesis I), followed by a period of accelerated production and more rapid buildup of ovarian mass (Vitellogenesis II) [39]. These two periods represent the major periods of energetic investment into reproduction by female crabs. The ovaries are not used as a location for general lipid storage, and consequently, during non-reproductive periods the gonad mass declines substantially (e.g., [40]). The allocation of lipids to the ovaries therefore demarcates the beginning of the reproductive process. Following this period of egg energetic provisioning, oviposition occurs as eggs are extruded through the spermatheca where they are fertilized [41]. Eggs are then deposited on the pleopods where they remain until hatching. Thus the primary period of energetic investment into reproduction by most female crabs occurs prior to oviposition during the period of vitellogenesis. Prior to this period, long-term energy reserves are built up primarily as lipid stores in the hepatopancreas, a digestive organ [42]. Consequently, I define capital breeding as breeding that draws on this store of lipids sequestered in the hepatopancreas prior to the start vitellogenesis in the ovaries, and income breeding as energetic investment that occurs after the initiation of vitellogenesis in the ovaries, but before oviposition.

Crabs have generally been considered to be capital breeders based on two pieces of evidence. First, proteins that are prominent in vitellogenesis are common prior to reproduction in the hepatopancreas [42]. Second, there is a decrease in lipid stores in the hepatopancreas at the same time that there is a buildup in the ovaries [43,44]. Based on this concept, numerous studies have demonstrated in various species an inverse correlation between the buildup of the ovaries throughout the reproductive process and the simultaneous diminution of the hepatopancreas (see numerous crab species listed in Table 1 of [45]).

Despite a general capital breeding paradigm for crustaceans, not all species display this inverse pattern in the size of the ovary and the size of the hepatopancreas. For instance, no correlation was found between the sizes of these two organs across individuals in the Asian shore crab *Hemigrapsus sanguineus* [46]. Additional evidence also suggests that crustaceans may be capable of using either an income strategy or a mixed capital-income breeding strategy. For instance, lipid accumulation in the ovaries is often greater than that stored in the hepatopancreas, suggesting that at least some lipid synthesis occurs directly in the ovaries during vitellogenesis [47]. Further, shrimp increase food consumption during lipid production, suggesting that lipid production during vitellogenesis derives directly from concurrent food consumption (i.e., income breeding) [48]. However, the ability of crabs to use an income breeding strategy was recently questioned [45].

Given the ecological and economic importance of this group of organisms in nearshore marine habitats around the globe, it is desirable to understand their responses to continued human-induced environmental changes. And determining their response to environmental change depends strongly on understanding their reproductive performance, which is controlled by their energetic strategy for financing reproduction. Unequivocal evidence in support of an income breeding strategy can only come from experimentation, where dietary intake is supplemented and/or controlled during egg production so that its influence on reproductive effort can be assessed directly [45]. Here I report such an experiment conducted with the Asian shore crab, *H*. *sanguineus*. I then use data from several similar experiments that have been previously published on a range of species from a broad phylogenetic sampling of crab families to assess whether each of them are capable of utilizing an income breeding strategy.

## Methods

### Asian shore crab feeding experiment

I collected 40 mature, female *Hemigrapsus sanguineus* (20.5±1.7 mm carapace width, mean±SD) from Odiorne Point State Park, NH on April 29, 2013, under a permit issued by New Hampshire Fish and Game. Only gravid crabs were selected. This species is capable of producing multiple clutches of eggs per year, with this process beginning in April [49]. Thus, the clutches of eggs being carried by these crabs were likely the first clutches of the reproductive season for each crab. Only using crabs that were gravid at the time of collection therefore standardized to the extent possible the physiological/energetic condition of each of the experimental crabs. Following collection, crabs were returned to the University of South Carolina where they were held for one week at elevated temperatures (20°C–which is within the range of summer time temperatures at the site where crabs were collected) to stimulate egg hatching. Crabs were not fed during this time, further standardizing their energetic condition.

Following this holding period, each crab was placed in a 1 L chamber that was then submerged in a recirculating water bath, held at 15°C (mimicking common summer coastal water temperatures on the New Hampshire coast), a salinity of 34 (using natural seawater collected from the South Carolina coast), and a 16 h:8 h light:dark cycle. Each of these experimental chambers was individually plumbed with a continuous supply of water from the recirculating system at a rate of 3 L h^-1^.

Crabs were fed twice weekly (Monday and Thursday) throughout the course of the 8-week experiment. Each of the 40 experimental crabs was randomly assigned one of 20 unique food treatments (described below). Thus, there were 2 crabs that were offered each of the 20 food treatments; however, these two crabs should not be considered experimental replicates. While it was possible to precisely control the amount and type of food offered throughout the experiment, the metric of interest was actual food consumed, not food offered, and food consumption was influenced by individual crab preferences and could not be experimentally controlled. Each of the 40 crabs therefore had a unique diet over the course of the experiment in terms of the total amount consumed and the proportion of consumed food that was animal vs. plant.

For each crab, food amount was based on the initial weight of the crab (no crabs molted during the experiment, and thus their weights were assumed to remain constant). Food treatments crossed four levels of food amount (determined based on wet weight of food as 3, 6, 12, and 24% of body weight at each feeding) and five levels of food type (0:1, 0.25:0.75, 0.5:0.5, 0.75:0.25, 1:0 ratio of animal:algae). The animal food used was tilapia filets. Asian shore crabs consume a range of animal prey [50], but in previous experiments with a natural diet of mussel tissue (*Mytilus edulis*), nonconsumptive loss of this tissue occurred due to degradation in the water [50]. Fish filets were used because they did not degrade in the same way and so allowed more precise determination of amount consumed. Tilapia and *M*. *edulis* are, however, similar in their energetic and nutrient content [51]. *Chondrus crispus* was used as the algal food, as this is a species commonly consumed by Asian shore crabs [52]. A nonconsumed control was included for both fish and algae at each feeding, which consisted of 1 g of each placed in a mesh bag in the same flow-through tank where the experiment was conducted. Crabs were given 24 h to eat the food provided, after which uneaten food was placed in a drying oven at 70°C for 48 h and then weighed. The initial dry weight of food offered was determined from the initial wet weight, the percent water content of each food type, and food loss via disintegration or leaching into the surrounding water, as determined using the nonconsumed controls. The amount of dry tissue of each food type consumed was then determined for each crab as the difference between the initial and final dry weight.

At the conclusion of the experiment, crabs were sacrificed by freezing, and were then dissected and the hepatopancreas, the ovaries, and the rest of the body were each dried separately for 72 h at 70°C, and then weighed. Energy storage and reproductive effort are commonly examined after controlling for body mass by dividing the mass of the ovary and hepatopancreas by the mass of the body (i.e., the gonadosomatic and hepatosomatic indices [53]). The goal of this study was to look for a positive correlation between reproductive effort and food consumption. In this case, using ratios, as with the gonadosomatic index, can yield a positive relationship between ovary and food consumption simply because both of these factors scale with body size [54,55]. Consequently, I analyzed the data using residual analysis to remove the effects of body mass. Specifically, I first performed linear regressions to determine the relationship between ovary mass and body mass, and between mass of food consumed and body mass. I then determined the residuals from each of these two regressions and used a linear model to examine the residual ovary mass as a function of the residual food consumption.

### Comparison across species

Several recent studies report results of feeding experiments with various crab species similar to the experiment described above for the Asian shore crab. None of these studies addressed income breeding, as each focused on other topics. These include experiments on two crabs of the family Portunidae, the European green crab *Carcinus maenas* [51] and the blue crab *Callinectes sapidus* [56], on *Cancer irroratus* within the family Cancridae [57], on *Panopeus herbstii* of the family Panopeidae [17], and on *Aratus pisonii* of the family Sesarmidae [58]. Each of these studies used a similar experimental design where crabs were fed for several weeks on a specific diet that varied the total amount of food offered to each crab and the proportion of food that was animal or algal tissue, and the precise amount consumed was quantified throughout. At the conclusion of each of these experiments, crabs were similarly dissected and the mass of their ovaries and the body mass were determined. For each of these five species, I therefore repeated the analysis described above for *H*. *sanguineus*.

## Results

**Fig 1:**
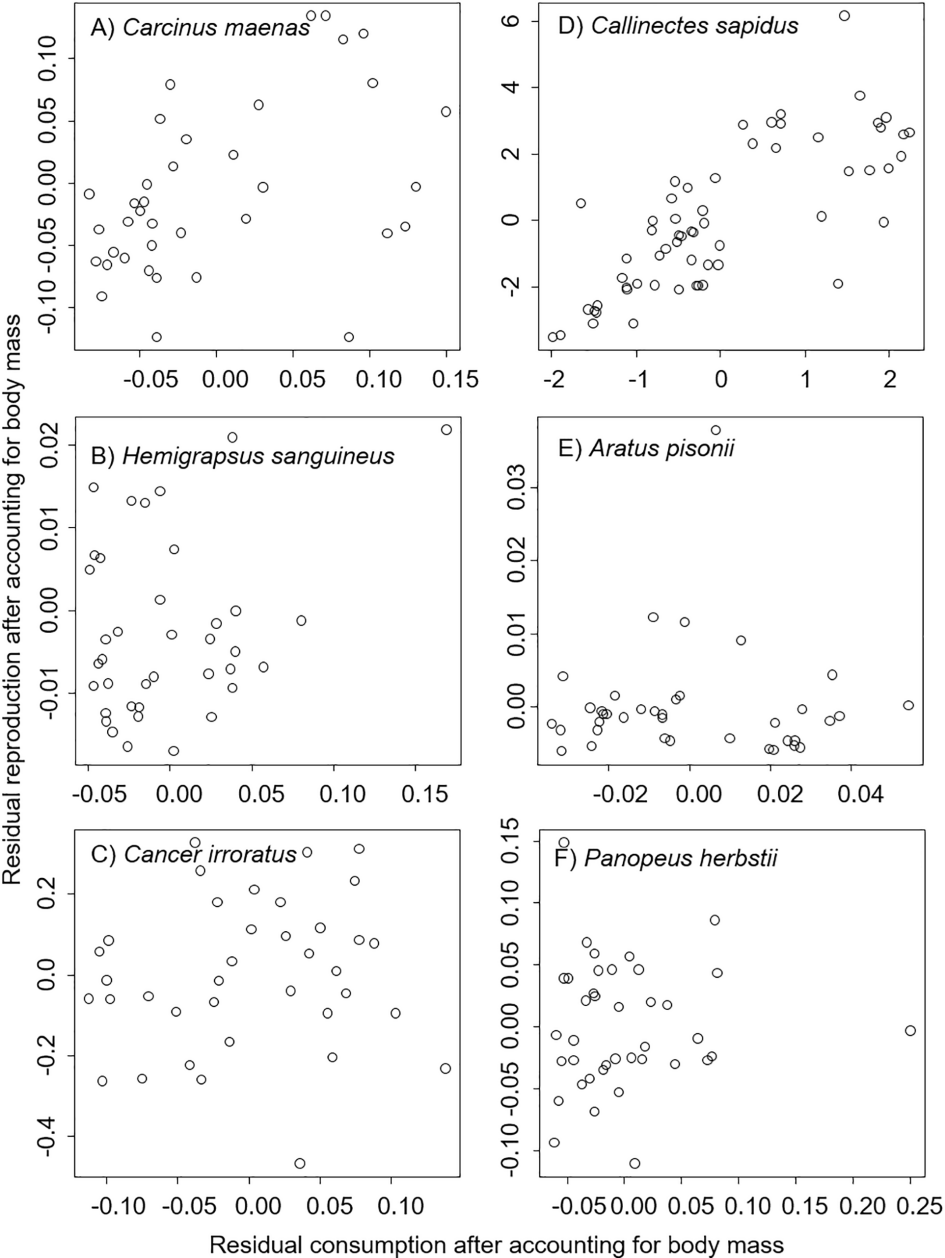
Relationship between residual ovary mass (after accounting for body mass) and residual food consumption (after accounting for body mass) for six species of brachyuran crabs. These results support the use of an income breeding strategy only for *Carcinus maenas* (A) and *Callinectes sapidus* (D).

Results here suggest species-specific use of income breeding among brachyuran crabs. Specifically, the residual analyses indicated that for both *C*. *maenas* (t = 3.10, *P* = 0.004, Fig 1A) and *C*. *sapidus* (t = 9.04, *P* < 0.001, Fig 1D), the residual ovary size increased with residual food consumption, supporting the use of an income breeding strategy. There was no positive relationship between residual reproductive effort and residual food consumption for *H*. *sanguineus* (t = 1.67, *P* = 0.11, Fig 1B), for *C*. *irroratus* (t = 0.72, *P* = 0.47, Fig 1C), for *A*. *pisonii* (t = 0.04, *P* = 0.97, Fig 1E), or for *P*. *herbstii* (t = 0.21, *P* = 0.84, Fig 1F), providing no evidence for income breeding in any of these species.

## Discussion

The results here provide clear evidence that, contrary to previous assumptions, some brachyuran crabs (*C*. *maenas* and *C*. *sapidus*) use an income breeding strategy. These species are both in the family Portunidae [59]. Studies on additional species are required before it is clear whether income breeding is phylogenetically determined. For two of these species (*C*. *irroratus* and *P*. *herbstii*) the failure to find evidence of income breeding here demonstrates conclusively that these species do not use income breeding. This is because each of these species produces only a single clutch of eggs per year [60,61]. However, each of the other two species examined (*H*. *sanguineus* and *A*. *pisonii*) produce multiple clutches of eggs annually [35,62]. It has previously been suggested that animals that produce multiple batches of offspring annually may produce the first batches using a capital strategy that utilizes energy stored prior to the reproductive season, with subsequent, lower quality batches being produced later in the reproductive season using income energy acquired during the reproductive season [63]. Patterns of declining egg quality in successive clutches in other species of brachyuran crabs (*Menippe mercenaria*, [64]) would seem to support this pattern. The experiment with *A*. *pisonii* used animals collected in June [58], towards the beginning of the reproductive season for Florida where crabs were collected [65]. Similarly, the experiment with *H*. *sanguineus* used animals collected in April, which is the beginning of the reproductive season for this species at the collection site. Thus, it is possible that if these experiments were repeated later in the reproductive season, after stored energy capital had been depleted, that an income strategy may be used. Thus, the lack of evidence here for income breeding in these two species does not demonstrate that they never use income breeding.

### Broader implications

I have demonstrated income breeding in *C*. *maenas*, but this species can also employ capital breeding [66]. This species has been a successful invader under a wide range of conditions [67], and the use of both capital and income breeding strategies may help explain some of this success. Both capital and income breeding strategies, on their own, have drawbacks. Population dynamics in income breeders are expected to be tied more closely to environmental fluctuations, and particularly to the availability of food during reproductive periods [11], while capital breeders incur the additional metabolic cost of maintaining energy stores [19,21]. The ability to use both of these strategies may help *C*. *maenas* to avoid/reduce each of these drawbacks in order to maximize success, as storing some capital across seasons ensures at least a minimum reproductive effort by individuals, while the ability to augment those stores with food consumed during the reproductive season allows individuals to take advantage of good years and/or high quality food patches to further increase their reproductive effort. This capability may in fact explain at least in part the documented high variation in reproductive performance between individuals within populations of this species [51].

As suggested previously [46], reproductive strategy and flexibility may influence invasion success in at least two additional ways. First, capital breeding may allow crabs to take advantage of carry-over effects if adults carrying capital from the source habitat are transported to the novel habitat [68]. Second, the extent to which individual animals are flexible in their ability to use a capital or an income strategy is not yet clear. If there is individual flexibility between these two strategies, this could potentially allow introduced individuals to succeed in novel environments that are phenologically different from their source environment in food provisioning or thermal periods (for instance, due to differences in latitude). For similar reasons, the ability to use a mixed capital-income strategy may help buffer crabs against a range of human-induced environmental stresses (climate change, habitat deterioration) that alter the availability or variability in food supply.

Finally, determining the mode and timing of energetically financing reproduction also has important implications for crab fisheries and aquaculture. For instance, claw-based fisheries, such as that for the Florida stone crabs *Menippe* spp. (not examined here), take only claws, returning captured crabs to the water alive where they will ideally regrow their claw(s) and re-enter the fishery, as well as continue to contribute to population growth. However, claw loss substantially reduces consumption rates and inflicts a direct metabolic cost related to claw regrowth [67–71]. The mode of financing reproduction, combined with the timing of claw loss relative to the reproductive season, therefore has important implications for the tradeoff between claw regrowth and reproductive effort [72]. In aquaculture, considerable effort has gone into optimizing food type [73,74] and feeding schedules for crab larvae [75,76]. Results here suggest that optimizing reproductive output of brood stocks may also depend on the timing of feeding adults.

### Predicting the consequences of capital and income breeding in crabs

It has previously been predicted that body size should be an important predictor of the ability to use a capital breeding strategy [11]. Body size is an important factor controlling the physiology of crabs because of the limited volume inside their carapace [77]. Carapace shape, and thus volume, varies across taxa, and taxa with smaller carapace volume often compensate for their inability to produce large clutches due to internal space limitation by producing multiple clutches of eggs per year [77]. Other ecological factors can also influence the physiology of crabs, altering the space occupied by vital organs, with implications for available space for energy storage. For instance, gill volume varies by a factor of three for a given size crab [78], being much larger for fully aquatic than for semiaquatic or primarily terrestrial species [79]. Additionally, stomach volume varies with diet, and for a given sized crab, species that are primarily herbivorous have guts that can be an order of magnitude larger than species that are primarily carnivorous [80]. Thus, the space available for storing energy during capital breeding should be most limited for aquatic herbivores from taxa that have relatively small carapace shape.

In summary, I have shown that income breeding does occur in some brachyuran crabs. Combined with previous evidence for capital breeding, this suggests that this ecologically and economically important group of marine consumers are mixed capital-income breeders, but that the relative importance of these two strategies differs across species. Further work is required to determine whether the use of income breeding is phylogenetically determined, and whether individual animals are capable of shifting between these two strategies.
